# Influence of Menopausal Hormone Therapy on Body Composition and Metabolic Parameters

**DOI:** 10.1089/biores.2019.0050

**Published:** 2020-03-17

**Authors:** Graciela B.C. Costa, Gláucia Carneiro, Luciana Umeda, Dolores Pardini, Maria Teresa Zanella

**Affiliations:** Division of Endocrinology, Department of Medicine, Universidade Federal de São Paulo, São Paulo, Brazil.

**Keywords:** body composition, cardiovascular risk, menopausal hormone therapy, metabolic syndrome

## Abstract

The loss of estrogen with menopause is associated with an increase in central fat. The objective of this study was to evaluate the effects of menopause hormone therapy (HT) on body composition and metabolic parameters in postmenopausal women. A prospective study was conducted among postmenopausal women from the Climacteric clinic, Universidade Federal de São Paulo. Thirty-two participants, median age 51 years, were included. Sixteen women were eligible to receive a low-dose continuous combined HT, containing 1 mg of E2 plus 0.125 mg of trimegestone for 6 months. The other 16 women remained in the control group. In the HT group, significant decreases from baseline were evident for the total cholesterol (TC) (*p* < 0.05) and LDL levels (*p* < 0.05). The HDL significantly decreased (*p* < 0.05). However, the TC/HDL ratio also decreased (*p* = 0.05). The parameters of body composition, after 6 months of HT, were maintained. In the control group, body mass index levels increased from baseline, however, with nonstatistically significant differences (*p* = 0.06). Analyzing the body composition showed a significant increase in the trunk body fat (*p* = 0.04), trunk region fat (*p* = 0.04), and total region fat (*p* = 0.03) after 6 months. In conclusion, the present study provides evidence that HT can stunt the increase in total body fat and prevent the shift from a more central fat distribution observed in early postmenopausal period.

## Introduction

Menopause signifies a critical endocrine and metabolic phase.^1^ Estrogen promotes the accumulation of gluteofemoral fat, and the loss of estrogen with menopause is associated with an increase in central fat.^2^ There is evidence from basic and preclinical work that disruption of estradiol signaling may accelerate fat accumulation, disproportionately in the abdominal area, with increased insulin resistance and dyslipidemia.^3^

Abnormal body fat distribution rather than overall adiposity (obesity) plays an important role in the development of various metabolic and endocrine diseases, including atherosclerosis, hypertension, diabetes mellitus, and hyperlipidemia, thus increasing the incidence of cardiovascular diseases (CVD) after menopause.^4,5^

Menopause hormone therapy (HT), as demonstrated by observational studies, can be effective not only for symptom relief, but also for reducing CVD.^6^ In a recent study, oral estradiol therapy was associated with less progression of subclinical atherosclerosis (measured as carotid artery intima-media thickness) than was placebo when therapy was initiated within 6 years after menopause.^7^ These results are consistent with results of other studies that have suggested that the effects of HT on CVD may depend on the timing of therapy initiation relative to menopause.^8^

In contrast, the Women's Health Initiative (WHI) trial did not confirm the efficacy of HT in reducing the overall CVD risk.^9^ However, the women in the WHI were also older and had longer postmenopausal histories compared with the women who usually undergo menopausal HT in clinical practice.^10^

The aim of the present study was to evaluate the effects of menopause HT on body composition and metabolic parameters related to cardiovascular risk in postmenopausal women.

## Methods

A prospective study that was not randomized and or controlled with a placebo, was conducted among postmenopausal women who were recruited from the Climacteric outpatient clinic, Discipline of Endocrinology, Universidade Federal de São Paulo.

The eligibility criteria were plasma gonadotropin and estradiol levels in the postmenopausal range as confirmed by our laboratory: follicle-stimulating hormone (FSH) >34 mUI/mL and estradiol (E2) <25 pg/mL.

The participants were not eligible if they reported one of the following criteria: time of menopause >5 years, age >55 years, history of CVD or diabetes mellitus, use of drugs that can interfere with the carbohydrate or lipid metabolism (within the last 6 months), tabagism, alteration of the thyroid-stimulating hormone (TSH), current use of menopause HT, or a history of oophorectomy/hysterectomy. Women with known contraindications for HT, such as thrombotic disorders or hormone-sensitive cancer (cancer of breast, colon, lung, endometrium, or ovaries), were ineligible for the study.

After inclusion, the patients were divided in two groups, HT group and control group. The groups were matched by age, weight, body mass index (BMI), and waist circumference (WC).

Thirty-two participants, median age 51 years, were included for the present study. Sixteen women were eligible to receive a low-dose continuous combined HT, containing 1 mg of E2 plus 0.125 mg of trimegestone (Totelle^®^, Wyeth *Indústria Farmacêutica* Ltda., Brazil) for 6 months, and the other 16 women, who did not receive the treatment, remained in the control group.

Follow-up visits were performed during 6 consecutive months of the study. Weight was measured in kilograms using an anthropometric digital scale (Filizola^®^) with a 150 kg capacity, and height was measured using the scale's fixed stadiometer, which was capable of measuring from 95 to 190 cm. Both measurements, which were used to calculate the BMI, were obtained with the patients wearing light clothing and no shoes. The BMI was calculated by dividing body mass in kilograms by the height in squared meters. WC was defined as the abdominal circumference at the midpoint between the iliac crest and lowest rib, as measured in centimeters using a tape and not compressing the skin.

Venous blood samples (12 h fasting) were collected to assess the serum levels of estradiol, FSH, TSH, high-density lipoprotein (HDL) cholesterol, low-density lipoprotein (LDL) cholesterol, triglyceride, total cholesterol (TC), glucose, and insulin in both groups at baseline and at 6 months of the study period.

Insulin resistance was estimated by homeostasis model assessment (HOMA), calculated using the following formula: fasting plasma insulin (μUI/mL) × fasting plasma glucose (mmol/L)/22.5.

The body composition was determined by dual-energy X-ray absorptiometry (DEXA) using a lunar DPX, and the parameters of arms, legs, trunk, and total body were obtained.

These variants were registered at baseline and after 6 months, in both groups.

This project was approved by the Ethics Committee of Universidade Federal de São Paulo/Hospital São Paulo. All participants provided written informed consent.

### Statistical analyses

All data were analyzed using the software Statistical Package for the Social Sciences (SPSS), version 19.0 (SPSS Inc., Chicago, IL).

The statistical analyses for the group comparisons included either a parametric independent samples *t*-test or a paired samples *t*-test, as indicated.

Spearman coefficient was used to determine the correlations between the different variables after 6 months.

The data are expressed as the mean ± standard deviation, and *p* < 0.05 was considered statistically significant.

## Results

The mean menopausal period was 2.7 years, with no significant differences among the groups. The baseline anthropometric and laboratorial measures did not differ between the groups, except the TC, HDL, and LDL cholesterol were higher in the treatment group at baseline, as shown in Table 1. After 6 months, the treatment group had higher estradiol levels than the control group (48.6 ± 37.1 ng/dL vs. 11.9 ± 6.4 ng/dL, *p* < 0.05).

**Table 1.** Patient Characteristics at Study Inclusion and After 6 Months of Observation

	Hormone therapy group, N = 16		Control group, N = 16	
	Baseline	6 Months	Baseline	6 Months
Age, years	50.8 ± 3.1	—	51.5 ± 4.5	—
Time of menopause, years	2.6 ± 1.6	—	2.8 ± 1.7	—
BMI, kg/m^2^	26.6 ± 5.5	26.4 ± 5.4	26.1 ± 3.7	26.5 ± 3.7
WC, cm	89.6 ± 13.6	89.0 ± 14.7	88.8 ± 8.3	89.5 ± 8.0
Total cholesterol, mg/dL	229.0 ± 31.8^*^	197 ± 27.8^**^	183.8 ± 33.0	185.7 ± 23.1
HDL cholesterol, mg/dL	64.4 ± 15^*^	60.3 ± 15.9^**^	49.1 ± 9.5	48.8 ± 10.8
LDL cholesterol, mg/dL	143.6 ± 29.7^*^	116.3 ± 26.2^**^	113.0 ± 31.4	110.8 ± 22.9
TC/HDL ratio	3.7 ± 0.6	3.4 ± 0.7^**^	3.9 ± 1.2	4.0 ± 1.1
Triglycerides, mg/dL	102.2 ± 34.3	103.1 ± 45.3	108.3 ± 53.2	129.9 ± 76.2
Glycemia, mg/dL	91.5 ± 7.6	93.2 ± 8.5	93.3 ± 8.5	96.9 ± 11.5
Insulin, *μ*UI/mL	5.0 ± 3.4	3.7 ± 2.0	5.5 ± 3.0	6.2 ± 3.4
HOMA-IR	± 0.8	0.8 ± 0.4	1.2 ± 0.6	1.3 ± 0.7
Estradiol	14 ± 5.7	48.6 ± 37.1^**^	15.6 ± 18	11.9 ± 6.4
Body composition				
Arms				
Fat mass (kg)	2.535	2.610	2.621	2.681
Lean mass (kg)	3.958	3.898	4.304	4.338
Region fat	32.4%	33.7%	35.5%	35.8%
Legs				
Fat mass (kg)	9.154	9.099	7.990	8.335
Lean mass (kg)	12.518	12.287	12.192	12.332
Region fat	38.7%	39.2%	37.4%	38.3%
Trunk				
Fat mass (kg)	12.294	12.676	12.212	12.716^**^
Lean mass (kg)	17.789	17.880	17.216	17.040
Region fat	37.8%	38.6%	40.1%	41.4%^**^
Total				
Fat mass (kg)	25.261	25.589	23.718	24.639
Lean mass (kg)	36.838	36.845	36.662	36.674
Body fat	37.3%	37.7%	37.4%	38.4%^**^

^*^ *p* < 0.05 versus the control group.

^**^ *p* < 0.05 versus the baseline values.

BMI, body mass index; HDL, high-density lipoprotein; HOMA-IR, homeostasis model assessment-insulin resistance; LDL, low-density lipoprotein; TC, total cholesterol; WC, waist circumference.

**FIG. 1.**
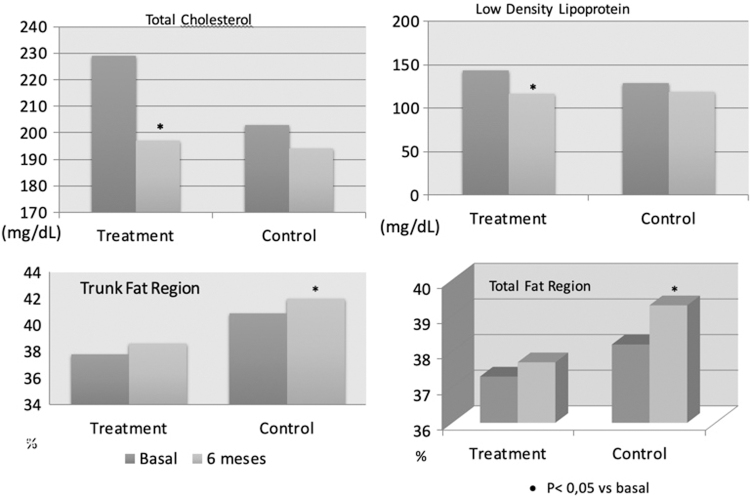
Dyslipidemic parameters and body composition in the two groups (Treatment and Control).

In the HT group, as demonstrated in Table 1 and Figure 1, significant decreases from baseline were evident for the TC (229 ± 31.8 mg/dL vs. 197 ± 27.8 mg/dL, *p* < 0.05) and LDL levels (143.6 ± 29.7 mg/dL vs. 116.3 ± 26.2 mg/dL, *p* < 0.05). The HDL significantly decreased (64.4 ± 15 mg/dL vs. 60.3 ± 15.9 mg/dL, *p* < 0.05). However, the TC/HDL ratio also decreased (3.7 ± 0.6 vs. 3.4 ± 0.7, *p* = 0.05) at the end of the study. No significant differences were found for BMI, WC, glycemia, insulin, HOMA-insulin resistance (HOMA-IR), and triglycerides after 6 months of HT. The estradiol levels showed a significant increase after 6 months of treatment (14 ng/dL vs. 48.6 ng/dL, *p* = 0.01), which was important to prove the adherence.

In the control group, compared with the baseline values, no changes were observed in WC, serum glucose, insulin, HOMA-IR, lipid profile, and estradiol after 6 months. Conversely, BMI levels increased from baseline, with no statistically significant differences (26.1 ± 3.7 kg/m^2^ vs. 26.5 ± 3.7 kg/m^2^, *p* = 0.06). The estradiol levels did not change (15.6 ng/dL vs. 11.9 ng/dL) (Table 1).

After analyzing the body composition after 6 months of HT, all parameters were maintained (Table 1), including the trunk body fat and total body fat. However, the control group showed a significant increase in the trunk body fat (12.212 ± 2.852 kg vs. 12.716 ± 2.810 kg, *p* = 0.04), trunk region fat (40.1 ± 6.3% vs. 41.4 ± 5.8%, *p* = 0.04), and total region fat (37.4% ± 5.4% vs. 38.4% ± 5.2%, *p* = 0.03) after 6 months. The other parameters were maintained (Table 1).

In the control group, we found a positive correlation between the changes in trunk region fat (*r* = 0.65, *p* = 0.029), total body fat (*r* = 0.75, *p* = 0.007), and HOMA-IR.

Considering all patients together, at the end of the study, the changes in serum estradiol showed a negative correlation with the changes in serum TC (*r* = −0.54, *p* = 0.008) and LDL cholesterol (*r* = −0.45, *p* = 0.032).

## Discussion

The results of this present prospective study showed that postmenopausal estrogen plus progestin therapy was associated with improved plasma lipid profiles. The study participants had lower TC and LDL levels and a lower TC/HDL ratio after 6 months of treatment.

The present results also confirm that the postmenopausal period, with no treatment, is associated with a significant body fat increase, particularly in trunk body fat, which was not evident in the treatment group. Postmenopausal women who received estrogen plus progestin during the study maintained their body composition parameters after 6 months of treatment.

Our study used the DEXA to estimate the total and regional body composition. It has the ability to show the regional distribution of body fat and provides good precision for body composition measurements. DEXA is more valid and precise than waist-to-hip circumferences ratio and less expensive and invasive than computerized tomography or magnetic resonance imaging.^11^

According to our results, Kristensen et al.^12^ found that HT attenuated the postmenopausal increment in fat mass by 60%. The reduction in fat accumulation was found on the trunk and, to a lesser extent, on the legs. This result aligns with studies^13,14^ in which the amount of adipose tissue was measured with DEXA scans, but the result contradicts other investigations.^15,16^ The controversy may relate to differences in the type and dosage of HT, the length of the observation periods, the number of study subjects, or the method used for estimating adipose tissue.

Ahtiainen et al. also published a study in which the authors used a monozygotic co-twin control design, including 10 twin pairs (56–62 years of age) discordant for HT (duration of HT, 2–10 years). In addition, 14 premenopausal women (29–35 years of age) who did not use HT were studied to evaluate the differences in metabolic health between the premenopausal and postmenopausal states. The study confirmed that long-term HT was associated with a healthier amount and distribution of body fat and better adipocytokine.^17^

Menopause transition and the loss of ovarian function is associated with atherogenic risk factors such as increases in fat mass and abdominal fat accumulation, dyslipidemia, elevated blood pressure, and proinflammatory and prothrombotic states. Influenced by low estrogen and high androgen levels, the hormonal alterations that occur during the menopausal transition contribute more to changes in the distribution of body fat than general obesity.^2^

The accumulation of abdominal fat is associated with increased insulin resistance,^18^ predisposing women to an increased risk for metabolic syndrome and CVDs. A recent study demonstrated that estradiol increases insulin-stimulated glucose disposal when administered to early postmenopausal women (within 6 years of menopause) compared with a decrease when administered to late postmenopausal women (>10 years past menopause).^19^ In our study, despite this significance, we observed that the HT group had lower fasting insulin levels and HOMA-IR, after 6 months. The opposite result was observed in the control group.

Previous studies have confirmed the role of abdominal fat mass and central obesity in the worsening of cardiovascular risks indices after menopause.^20^

Data from observational and randomized studies suggest that HT started soon after the menopause may be effective, not only for symptom relief, but also for providing cardiovascular benefits.^21–23^

Conversely, the WHI, a large randomized controlled trial that examined the risks and benefits of conjugated equine estrogens and medroxyprogesterone acetate in healthy women,^9^ did not confirm the efficacy of HT in reducing the overall CVD risk. The treatment duration and timing of menopausal HT initiation seem to be distinct factors that may help explain the disparity in coronary heart disease outcomes between the observational studies and the WHI randomized trials.^10^ In addition, different types of HT and dose variations may affect the CVD risk differently.

In a review of the literature^24^ demonstrated that estrogens administered in the perimenopausal transition or early in menopause are not harmful to the cardiovascular system and, when administered for a few years to treat menopausal symptoms, may slow the progression of atherosclerosis and reduce the postmenopausal CVD risk.

The decisions to prescribe menopausal HT and how long to continue their administration should be flexible and based on patient characteristics and the balance of benefits and risks.

As mentioned on the 2017 HT position statement of The North American Menopause Society, HT may help attenuate abdominal adipose accumulation and the weight gains that are often associated with the menopause transition. The present study provides evidence that HT can stunt the increase in total body fat and prevent the shift from a more central fat distribution observed in normal women throughout the early postmenopausal period. In addition, our study confirmed the benefits of HT on the lipid profile. The strength of our study is a prospective cohort without drop-off during the 6-month follow-up.

One limitation of this study is the absence of a control group using placebo and lack of randomization. Also, the sample size is small and it was a convenience sample, not representative, which may limit the applicability of our results.

## Conclusion

Our results indicate that new clinical trials are needed to evaluate the influences of timing, duration, dose, administration route, and choice of agents on HT to optimize the recommendations for individual patients.

## Abbreviations Used

BMI: body mass index
CVD: cardiovascular diseases
DEXA: dual-energy X-ray absorptiometry
FSH: follicle-stimulating hormone
HDL: high-density lipoprotein cholesterol
HOMA-IR: homeostasis model assessment-insulin resistance
HT: hormone therapy
LDL: low-density lipoprotein cholesterol
SD: standard deviation
TC: total cholesterol
TSH: thyroid-stimulating hormone
WC: Waist circumference
WHI: Women's Health Initiative
